# Common and Unique Transcription Signatures of YAP and TAZ in Gastric Cancer Cells

**DOI:** 10.3390/cancers12123667

**Published:** 2020-12-07

**Authors:** Yaelim Lee, Megan Finch-Edmondson, Hamizah Cognart, Bowen Zhu, Haiwei Song, Boon Chuan Low, Marius Sudol

**Affiliations:** 1 Mechanobiology Institute, National University of Singapore, Singapore 117411, Singapore; mbihaa@nus.edu.sg (H.C.); dbslowbc@nus.edu.sg (B.C.L.); marius.sudol@mssm.edu (M.S.); 2 Genome Institute of Singapore, Agency for Science, Technology and Research, Singapore 138672, Singapore; Zhu_Bowen@gis.a-star.edu.sg; 3 Department of Physiology, National University of Singapore, Singapore 117593, Singapore; 4 Institute of Molecular and Cell Biology, Agency for Science, Technology and Research, Singapore 138673, Singapore; haiwei@imcb.a-star.edu.sg; 5 Department of Biological Sciences, National University of Singapore, Singapore 117543, Singapore

**Keywords:** YAP (Yes-associated protein 1, also known as YAP1 or YAP65), TAZ (transcriptional co-activator with PDZ-binding motif, also known as WWTR1), gastric cancer, transcriptome, platelet, lipoprotein particles, cell-substrate junctions

## Abstract

**Simple Summary:**

*YAP* and *TAZ* are cancer-causing genes that encode proteins with similar, but not identical functions. YAP and TAZ function in diverse biological processes including cell proliferation and organ size control. Because of the high similarity in functions between YAP and TAZ, they have often been described as one entity: YAP/TAZ. However, new lines of evidence started to suggest that YAP and TAZ have unique functions as well. To understand the YAP- and TAZ-specific functions, we identified genes that are regulated solely by YAP or by TAZ. Our study revealed that YAP plays a distinct role in cell-substrate junctions, which are critical for tumour cell growth, migration, and metastasis, and both YAP and TAZ are involved in regulating blood platelets and lipid metabolism in gastric cancer cells.

**Abstract:**

YAP and its paralog TAZ are the nuclear effectors of the Hippo tumour-suppressor pathway, and function as transcriptional co-activators to control gene expression in response to mechanical cues. To identify both common and unique transcriptional targets of YAP and TAZ in gastric cancer cells, we carried out RNA-sequencing analysis of overexpressed YAP or TAZ in the corresponding paralogous gene-knockouts (KOs), TAZ KO or YAP KO, respectively. Gene Ontology (GO) analysis of the YAP/TAZ-transcriptional targets revealed activation of genes involved in platelet biology and lipoprotein particle formation as targets that are common for both YAP and TAZ. However, the GO terms for cell-substrate junction were a unique function of YAP. Further, we found that YAP was indispensable for the gastric cancer cells to re-establish cell-substrate junctions on a rigid surface following prolonged culture on a soft substrate. Collectively, our study not only identifies common and unique transcriptional signatures of YAP and TAZ in gastric cancer cells but also reveals a dominant role for YAP over TAZ in the control of cell-substrate adhesion.

## 1. Introduction

YAP and its paralogous protein, TAZ, are nuclear effectors of the Hippo signalling pathway that controls organ size by regulating the balance between cell proliferation and apoptosis [1]. When deregulated, amplified, or fused to genes encoding transcription factors, YAP and TAZ can act as potent oncogenes [2]. Although YAP and TAZ proteins are fairly similar to each other in terms of their modular structures, functional domains and conserved regulatory phosphorylation sites [3], the phenotypes of individual knockouts (KOs) in mice differ significantly [4,5,6], suggesting non-redundant functions. 

Recently, YAP and TAZ have been actively investigated with regard to their roles in mechanobiology [7]. YAP/TAZ are frequently referred to as mechano-responders because of their ability to translocate into the nucleus to activate the transcriptional program in response to various mechanical cues, such as stiffness of substrate, cell stretching, fluid friction stress, or changes in the size of the adhesive area [7]. These biophysical signals are often altered in the tumour microenvironment, which closely interacts with cancer cells, leading to abnormal cellular responses such as proliferation and invasion [8,9]. In line with this, YAP and TAZ are up-regulated in multiple cancers, including gastric cancer where YAP/TAZ play a role in tumour growth [10]. Nevertheless, the transcriptional program controlled by YAP and TAZ in gastric cancer cells has not been investigated. Moreover, it is still not clear if YAP and TAZ play redundant or specific roles in mechanotransduction. 

In this study, we identified comprehensive transcriptional targets regulated by YAP and TAZ in gastric cancer cells via RNA-sequencing (RNA-seq). Specifically, we first generated YAP KO and TAZ KO cells using the CRISPR/Cas9 system, then subsequently overexpressed TAZ in YAP KO cells and YAP in TAZ KO cells to interrogate solely YAP or TAZ effects on transcription in the absence of its paralog. As a result, we identified common YAP/TAZ transcription targets in gastric cancer cells, which function in platelet biology and lipoprotein particles assembly. Interestingly, we uncovered that the cell-substrate junction and focal adhesion (FA) GO terms were enriched specifically in YAP transcriptional targets, indicating the unique function of YAP in activating the expression of cell-substrate junction components. To validate our transcriptome analysis, we investigated cell adhesion and spreading using YAP KO, TAZ KO, and wild type (WT) cells. We found that YAP KO cells had significantly impaired cell-substrate junction, particularly when the cells experienced a change in substrate rigidity.

## 2. Results

### 2.1. Generation of RNA-seq Data to Examine YAP and TAZ Effects on Transcription in Gastric Cancer MKN28 Cells

**Figure 1:**
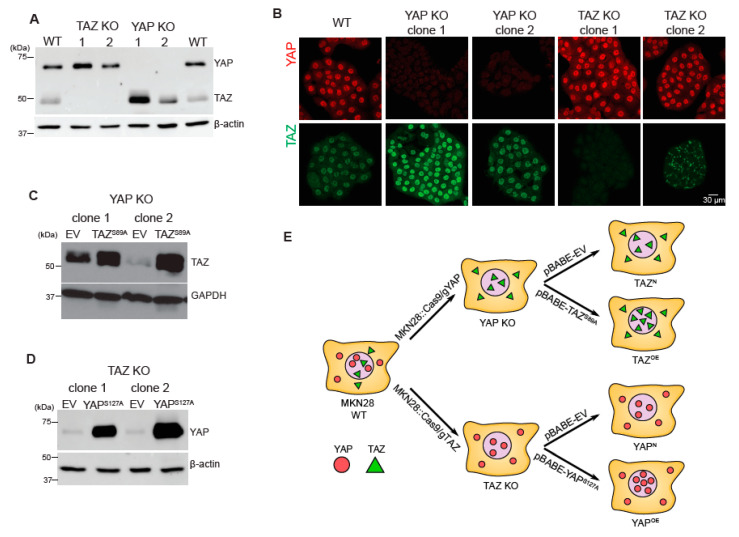
RNA-sequencing (RNA-seq) of the samples generated with gastric cancer MKN28 cells and activated mutants of YAP or TAZ genes. (**A**) Western blot analysis of YAP knockout (KO) and TAZ KO clones generated via the CRISPR/Cas9 system using two independent guide RNA sequences (labelled as 1 and 2) against human YAP and human TAZ. The wild type (WT) sample is shown in the first and last lane. β-actin is used as a loading control. The protein molecular weight size marker (unit: kDa) is depicted along the left of the Western blot images. The uncropped western blot figures in Figure S1. (**B**) Representative immunofluorescence images of YAP and TAZ in WT, two clones of YAP KO and TAZ KO. (**C**) Western blot analysis of YAP KO cells transduced with empty vector (EV, control) or TAZ^S89A^ (overexpression, OE). GAPDH is used as a loading control. (**D**) Western blot analysis of TAZ KO cells transduced with empty vector (EV) or YAP^S127A^ (OE). β-actin is used as a loading control. The uncropped western blot figures in Figure S2. (**E**) A schematic diagram of the samples prepared for RNA-seq.

To interrogate the common and distinct transcripts regulated by YAP and TAZ at the genome-wide level, we used a gastric cancer cell line, MKN28 [11]. We first generated two independent YAP KO and two independent TAZ KO clones using the CRISPR/Cas9 system utilising different guide RNAs to minimize the possibility of the off-target effects (Figure 1A,B). As the abundance of TAZ is affected by the loss of YAP [12], which may cause confounding effects on gene expression, we decided to use the respective KO cells as the baseline for measuring YAP- or TAZ-dependent gene expression. Subsequently, we overexpressed YAP in TAZ KO cells by stably-overexpressing a hyperactive YAP mutant construct, YAP^S127A^ (Figure 1D) to identify transcription targets controlled by YAP. Mutation of YAP that changes its Serine 127 to Alanine prevents YAP protein from being phosphorylated by LATS1/2 kinases, and from cytoplasmic retention and subsequent degradation [13]. As a result, YAP is accumulated in the nucleus leading to the activation of its target genes [13,14]. Likewise, we utilized the TAZ^S89A^ mutant [14,15] to enhance TAZ activity in YAP KO cells to assess TAZ-dependent transcriptional regulation (Figure 1C). Empty vector-transduced cells (EV) were generated for each YAP KO and TAZ KO clone to act as the respective control. An overview of our methodology is provided in Figure 1E. RNA-seq analysis was performed on the endogenous TAZ (EV control cells, named as TAZ^N^ hereafter) and overexpressed TAZ (TAZ^OE^) in YAP KO cells, and endogenous YAP (EV control cells, YAP^N^) and overexpressed YAP (YAP^OE^) in TAZ KO cells (Figure 1E).

### 2.2. Comprehensive Identification of YAP- and TAZ-Transcriptional Targets in Human Gastric Cancer MKN28 Cells

**Figure 2:**
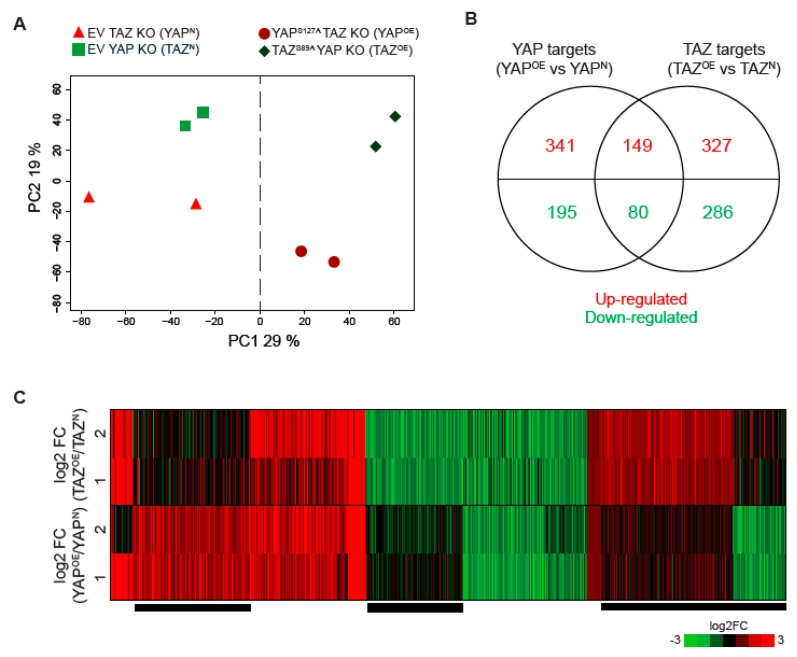
YAP- and TAZ-regulated transcriptional targets. (**A**) The PC1 vs. PC2 plot from the Principal component analysis (PCA) on the RNA-seq data using NOI-seq R package [17]. (**B**) A Venn diagram showing the numbers of transcripts that were up- or down-regulated by YAP overexpression in TAZ KO cells (left circle in the Venn diagram, YAP targets) and TAZ overexpression in YAP KO cells (right circle in the Venn diagram, TAZ targets). (**C**) A heatmap of k-means clustering of the differentially expressed transcripts (DETs) which are the union of YAP and TAZ targets. Each row corresponds to a log_2_ fold change (FC) of overexpression (OE) with respect to endogenous (N) samples, and each column corresponds to a DET. Numeric row names, 1 and 2, indicate respective KO clone 1 and clone 2. Red and green colour indicates activated and repressed transcripts in both (**B**) and (**C**). Black lines along the bottom indicate a subset of DETs showing distinct expression patterns between TAZ- and YAP-regulated transcripts.

We first checked the RNA-seq data quality by Principle Component Analysis (PCA), and this confirmed that the first Principal Component (PC1) correlated well with YAP/TAZ activity, which is our primary treatment, and the replicates were clustered together (Figure 2A), ensuring the reliability of the dataset.

To define the differentially expressed transcripts (DETs), we applied a threshold if both biological replicates showed an absolute fold difference greater than 2 in the overexpression sample compared to the respective control (i.e., |log2$\frac{OE\left(KOclone1\right)}{N\left(KOclone1\right)}$| > 1 and |log2$\frac{OE\left(KOclone2\right)}{N\left(KOclone2\right)}$| > 1) to ensure a consistent change in expression between the replicates. As a result, we found 817 up-regulated and 561 down-regulated transcripts mediated by YAP and TAZ in gastric cancer cells (Figure 2B). A list of transcripts showing a high fold difference are shown in Table S1. Known YAP/TAZ targets including *ANKRD1*, *CYR61*, and *CTGF* were included in the top up-regulated transcripts, indicating that our experimental design worked as expected. 

To investigate how similar or dissimilar the transcriptional response to YAP or TAZ activation was, we performed the k-means clustering analysis with all 1378 DETs (Figure 2C) using Cluster3.0 [16]. We observed approximately half of the DETs revealed a similar expression change upon YAP- and TAZ-overexpression, indicating the redundant role of YAP and TAZ in transcription. Contrastingly, another half of the DETs showed a distinct expression pattern between YAP- and TAZ-overexpression, suggesting unique transcriptional targets of YAP and TAZ (depicted as black lines along the bottom in Figure 2C).

### 2.3. Common and Unique Transcription Signatures of YAP and TAZ in Gastric Cancer Cells

**Figure 3:**
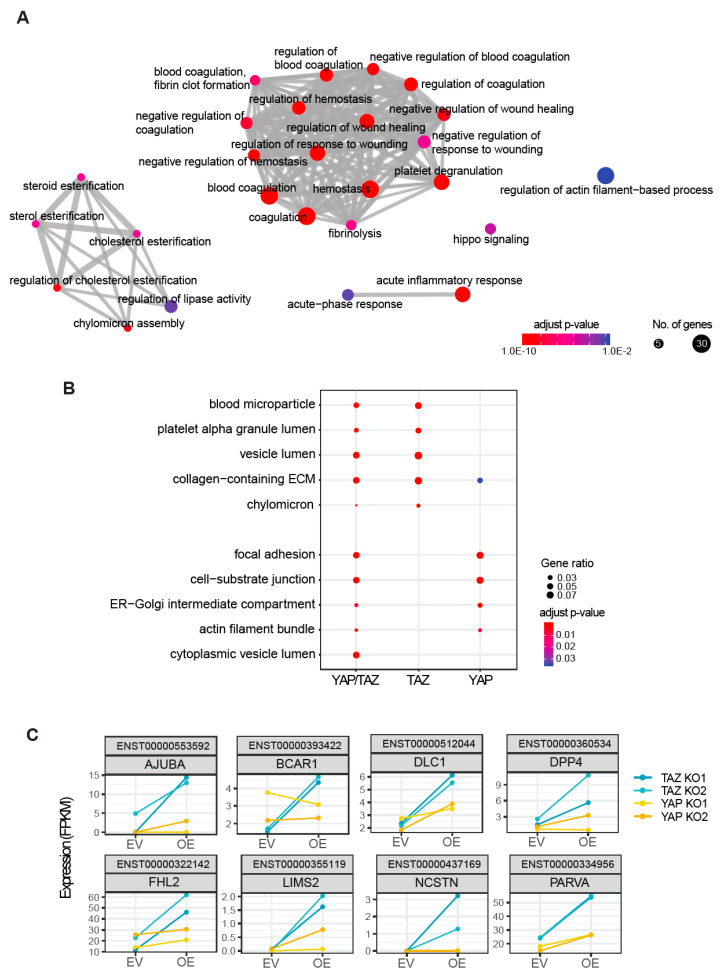
Gene Ontology (GO) analysis of YAP- and TAZ-transcriptional targets. (**A**) Biological process GO terms that are significantly enriched for the union of upregulated YAP targets and TAZ targets. Each GO term is depicted as a node (i.e., dot) and one node is connected to another node by an edge (i.e., line) if there is a significant overlap between the gene sets. The node size corresponds to the number of genes in the set and the node colour indicates the statistical significance of a GO term enrichment test. (**B**) The most significant cellular component GO terms for the up-regulated genes by YAP/TAZ (the union of YAP and TAZ), TAZ, and YAP. The dot size indicates a gene ratio, the number of genes in the input list associated with the given GO term, and then divided it by the total number of input genes. The dot colour depicts the statistical significance of a GO term enrichment test. (**C**) Representative genes assigned to the YAP-specific GO terms: focal adhesion (FA) and cell-substrate junction. Each box shows the expression level of a gene, measured by RNA-seq. The respective Ensembl transcript ID and gene symbol are shown on the top of each box. The Y-axis is expression level in Fragments Per Kilobase Million (FPKM). The X-axis indicates control and treatment groups: empty vector (EV) and overexpression (OE). Two TAZ KO clones and two YAP KO clones are depicted in blue and ochre, respectively. In other words, EV and OE in TAZ KO clones (blue) indicates YAP^N^ and YAP^OE^, and EV and OE in YAP KO clones (ochre) means TAZ^N^ and TAZ^OE^.

We next assessed the possible functions of YAP and TAZ in gastric cancer cells by conducting GO analysis on the DETs. Given that YAP and TAZ are transcriptional co-activators that bind primarily to individual members of TEAD transcription factors [18] to induce target gene expression, we first performed GO analysis of up-regulated genes (DETs in red in Figure 2B). To visualize the GO analysis result, we generated a GO enrichment map in which GO terms with overlapping gene sets are connected by an edge, forming a cluster [19]. For the union of YAP targets and TAZ targets, five separate clusters of GO terms were significantly enriched (Figure 3A), the largest cluster being wound healing-related processes, interconnected with the regulation of haemostasis, platelet degranulation and blood coagulation (Figure 3A). The second-largest cluster was lipoprotein-related terms, including regulation of cholesterol esterification and chylomicron assembly (Figure 3A). The GO analysis regarding cellular component terms with the same set of genes revealed consistent results, including blood microparticle, platelet alpha granule, and chylomicron (YAP/TAZ column in Figure 3B). In sum, our data show the existence of YAP/TAZ-dependent transcriptional signatures in gastric cancer cells, which is the activation of genes involved in platelets and lipoproteins.

Next, we questioned the unique functions of YAP and TAZ by comparing the significantly enriched GO terms for the YAP targets and TAZ targets, separately. We found that blood microparticle, platelet alpha granule lumen, and chylomicron GO terms were preferentially enriched in TAZ-targets, whereas focal adhesion (FA) and cell-substrate junction were specifically enriched in YAP-targets, indicating a functional divergence between YAP and TAZ (Figure 3B). 

Then, we interrogated genes that were assigned to every GO term in the GO analysis (Figure 3B) to confirm the functional difference between YAP and TAZ. This investigation indicated that many of the genes attributed to the TAZ-specific functions were also activated by YAP, but one of the biological replicates failed to pass the threshold that we applied to define DETs due to a possible batch effect, as indicated earlier in the PCA analysis (TAZ KO clone 1 and clone 2 transduced with empty vector, depicted as red triangles, were relatively distant in Figure 2A). This suggests that the seemingly TAZ-specific GO terms (TAZ column in Figure 3B) were highly likely to be YAP/TAZ common transcriptional signatures. However, the genes assigned to the YAP-specific GO terms—namely FA and cell-substrate junction—included 25 genes in common, 8 of which were highly up-regulated by YAP overexpression in TAZ KO cells (blue lines in Figure 3C). These 8 genes are *AJUBA* (Ajuba LIM Protein), *BCAR1* (also known as p130cas, Breast cancer anti-estrogen resistance protein 1), *DLC1* (Rho GTPase activating protein), *DPP4* (Dipeptidyl peptidase-4), *FHL2* (Four and a half LIM Domains 2), *LIMS2* (LIM zinc finger domain containing 2), *NCSTN* (Nicastrin), and *PARVA* (Parvin alpha). The expression of these genes was further validated in YAP KO in comparison of WT cells by RT-qPCR assay, and indeed the cell-substrate adhesion genes showed a significant reduction in expression for YAP KO cells compared to WT cells (Figure S5). Moreover, quantification of phosphorylated Paxillin at Tyr118, as an indicator of active FAs, using confocal microscopy revealed that the mean volume of the individual FAs in YAP KO cells was significantly reduced compared to those in WT cells (Figure S6). Collectively, the GO bioinformatics analysis, manual inspection of the individual genes assigned to the significant GO terms, and experimental validation via RT-qPCR and confocal microscopy suggested that YAP plays an important role in regulating cell-substrate junctions in gastric cancer cells.

### 2.4. YAP Is Indispensable for Adhesion to the Cell Substrate upon Altering Its Rigidity

Given our transcriptome analysis suggested that YAP, but not TAZ, has a role in activating cell-substrate junction genes, we hypothesized that YAP KO cells would show a defect in cellular behaviours that require the cell-substrate interaction, such as cell attachment and spreading when compared to TAZ KO and WT cells. First, we observed YAP KO, TAZ KO and WT cells from initial seeding to fully-spread stages in the culture dish using time-lapse live-cell imaging to see whether there was any deficiency in cell spreading in the absence of YAP. Yet, YAP KO cells started to spread on the culture dish without any noticeable delay and fully spread at a comparable rate to TAZ KO and WT cells (Video S1 https://drive.google.com/file/d/1i_fIO_gFsCmDhBg25CoFD8gj7obwDaM9/view; https://drive.google.com/file/d/1BC-OsRfhSer-3fabnb8IpRxNvuL3cgvR/view). 

Because cell attachment and spread are initiated by cellular probing of substrate rigidity, leading to cell proliferation and survival [20], we tested whether YAP KO cells showed a distinct cellular phenotype, compared to TAZ KO and WT cells, when exposed to different rigidities (soft and stiff). All cell lines grew well on the various rigidities tested, including 0.5 kPa, 2 kPa, 4 kPa, 50 kPa, and 100 kPa polyacrylamide (PAA) hydrogel dishes. There was no appreciable impairment in the proliferation of the KO cells compared to WT, except for YAP KO clone 2, which exhibited the lowest abundance of YAP and TAZ (Figure 1A,B). To ensure that the different proliferative capacity of the cell lines was not confounding our results, we utilised YAP KO clone 1 (YAP KO1) for subsequent rigidity experiments. 

**Figure 4:**
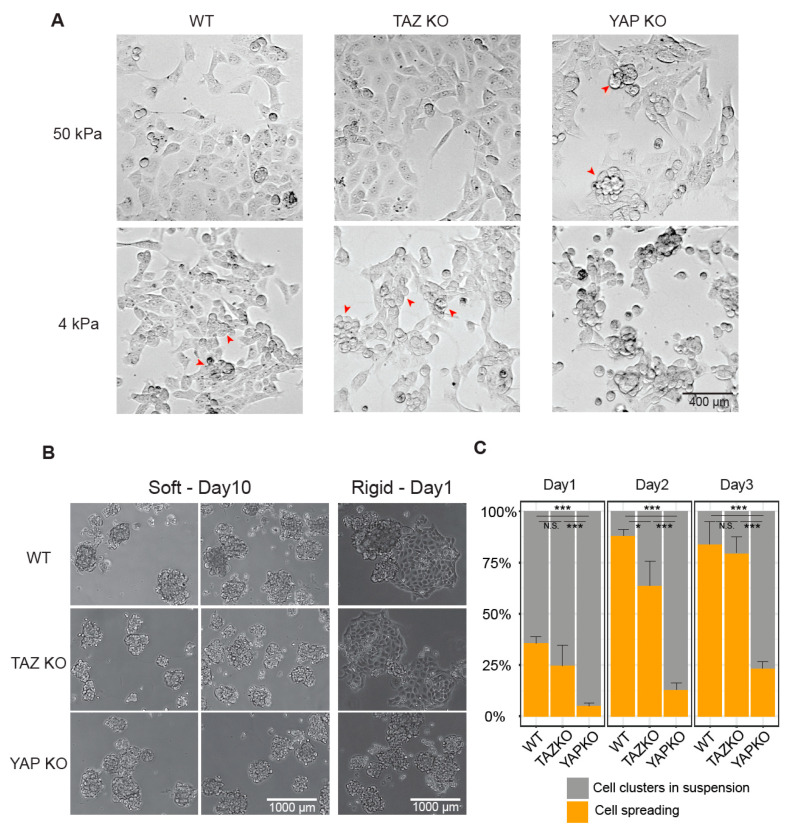
Wildtype (WT), TAZ knockout (KO), and YAP KO in different rigidity and in response to altering rigidity. (**A**) Representative images of WT, TAZ KO, and YAP KO cells cultured in polyacrylamide (PAA) hydrogel dishes with soft (4 kPa) and stiff (50 kPa) rigidity. (**B**) Representative images of WT, TAZ KO, and YAP KO cells maintained in 0.5 kPa PAA hydrogel dish for 10 days; and 1 day after transferring the cells to the rigid plastic dish. (**C**) Quantification of cells either attached (ochre) or in suspension (grey) on days 1, 2, and 3 after transferring cells to the rigid conditions. Quantification was done by visual inspection of 6–10 fields of view of phase-contrast microscopy per sample. Error bars indicate standard error of the mean, calculated from three biological replicates. The statistical significance level via two-tailed unpaired Student’s *t*-test was summarized with asterisks: *p* < 0.001 (***), *p* < 0.05 (*), and *p* > 0.05 (not significant, N.S.).

An obvious change in cell shape was observed when YAP KO, TAZ KO, and WT cells were grown on different rigidities, specifically, cells appeared as a simple squamous sheet on the stiff substrate (e.g., 50 kPa) and as 3D-cell clusters with marginal attachment to the soft substrate (e.g., 0.5 kPa) (Figure 4A,B). Interestingly, we noticed that the YAP KO cells in 50 kPa showed clusters of circular-shaped cells that were not attached to the substrate itself but interacted with the cells spread on the substrate (red arrows in Figure 4A), suggesting weaker cell-substrate attachment. However, no unattached cell clusters were shown with the TAZ KO and WT cells under 50 kPa conditions, rather this phenomenon was observed in softer conditions, 4 kPa, for the TAZ KO and WT cells. We also confirmed the weaker cell-substrate interaction particularly in 4 kPa for the YAP-null EpH4 cells, which were generated in our previous work [21], by detecting focal adhesion kinase phosphorylated at Tyr397, and Paxillin phosphorylated at Tyr118, both of which are indicative of active FAs, via Western blot (Figure S7). This indicates that YAP KO cells were less effective in forming the cell-substrate junctions when the cells were challenged by the softer rigidity than the typical laboratory culture conditions, such as plastic or glass-bottom dishes.

As the molecular composition of the cell-substrate junctions, such as FAs, is dynamically regulated in response to extracellular cues [22], we, therefore, hypothesized that YAP’s role in mediating cell-substrate junctions may become more apparent under altered substrate conditions rather than the constant state. Hence, we examined the cellular behaviour of YAP KO cells compared to TAZ KO and WT cells upon altering substrate rigidity. Specifically, we maintained the cells in the soft conditions (0.5 kPa) for a prolonged period of time (e.g., 10–13 days) to adjust an environment where the cell-substrate junction is a minimal contributor to cell growth (Figure 4B). We then transferred the cells to rigid dishes (plastic and glass) (Figure 4B) where the cells normally form many FAs (Figure S8). After transferring to the rigid conditions, approximately 25~35% of TAZ KO and WT cells were successfully attached to the culture dish and fully spread within 24 h, with the majority of cells (>75%) spreading within 72 h (Figure 4C). Notably, however, YAP KO cells showed a striking impairment in cell attachment and spreading upon transfer to the rigid conditions. Circa ~ 5% at first and then only 20% of the cells were attached to the culture dish in 24 and 72 h, respectively (Figure 4C). This data suggests that YAP is essential for cell-substrate attachment in response to changing substrate rigidity. 

## 3. Discussion

Using a model of gastric cancer cells and gene-editing approach, combined with targeted overexpression of active forms of YAP and TAZ oncoproteins, we identified a set of their common target genes. These genes are primarily involved in platelet biology and lipoprotein particle formation. The interplay between platelets and lipoproteins has been studied extensively in atherosclerosis because lipoprotein particles trigger the platelet signalling pathway by binding specific receptors on the platelet [23], contributing to atherosclerosis via multiple mechanisms [24]. In addition, the similarities between atherosclerosis and cancer at a molecular level, and parallels of cellular phenotypes of uncontrolled proliferation, chronic inflammation and thrombosis could perhaps be explained in part by the gene signatures of YAP and TAZ [9,25,26,27]. Furthermore, the disturbed blood flow, manifested in atherosclerosis, generates mechanical forces, that could lead to YAP/TAZ activation [28,29]. Our finding that YAP and TAZ activate platelet- and lipoprotein-genes transcriptionally may corroborate the functional association between the common players in atherosclerosis/cancer pathophysiology.

Also, we uncovered the preferential function of YAP in up-regulating the expression of genes that encode proteins involved in cell-to-substrate adhesion. A recent study [30] demonstrated that focal adhesion genes are direct transcription targets of YAP by chromatin immunoprecipitation (ChIP)-sequencing analysis. Moreover, another study in which the effect of fluid shear stress on cell motility was tested in prostate cancer cells showed that YAP knockdown, but not TAZ knockdown, abolished the enhanced cell migration induced by fluid wall shear stress in prostate cancer cells, underscoring distinctive roles of YAP and TAZ in response to this mechanical stimulus [31]. Our observation that the cell-substrate junction genes are significantly enriched in YAP transcription targets provides additional evidence to support the notion that YAP could be a dominant player governing mechanotransduction.

Cancer cells display resistance to anoikis, a programmed cell death that is induced upon cell detachment from the extracellular matrix [32]. Upon departing from the primary tumour site, cancer cells then proliferate and survive in the blood or lymphatic systems representing metastatic seeding to secondary sites [33,34]. Indeed, anoikis was shown to be regulated by YAP and TAZ [7,35], and one of the mechanisms by which cancer cells achieve resistance to anoikis is via physical association with platelets in the blood [9,36]. Changes in the rigidity of tumour stroma are known to contribute to the metastatic potential of cancer cells [8]. Therefore, we implemented here an experimental set-up in which we applied substrata of altering rigidity to the cancer cells, and this challenge revealed a striking impairment of YAP KO cells in spreading onto the new rigidity settings. This relatively simple experiment unveiled yet another facet of the potential role that YAP may play in cancer metastasis [21].

We are fully aware of several limitations and weak points of this report, which include the use of only one kind of cell line, namely the MKN28 gastric cancer cells in the transcriptomic analysis, which may represent a particular subset of gastric cancer. We cannot exclude a possibility that certain transcripts that we identified as YAP and TAZ signature genes could be cell line-specific transcripts. Although we were careful with the CRISPR/Cas9 gene-editing approach by using two different RNA guides with which we generated two different clone isolates for each YAP KO and TAZ KO, we did not perform a rescue experiment on these lines with wild type YAP and TAZ, followed by the RNA-seq analysis. Usually in overexpression experiments, one cannot be fully confident if the determined transcriptome reflects precisely the actual biology or if it is partly skewed by over-saturation with the activated transgene that encodes a transcriptional regulator. For each of the YAP and TAZ targets that were identified in our screen, one ought to perform ChIP-sequencing analysis to increase confidence about the direct transcriptional targets of YAP and TAZ. Moreover, we used only one of the eight splice isoforms of *YAP* [37] as a cDNA clone to express in TAZ KO cells for the transcriptomic analysis and our *TAZ* cDNA for expression on YAP KO human cells was a mouse homolog. The mouse TAZ is 91% identical in the amino acid sequence to the human TAZ protein, with 24 similar substitutions between the two proteins. Despite these weaknesses, we believe that our data makes an important and original contribution to the discussion of how YAP and TAZ signal as mechanotransducers.

## 4. Materials and Methods 

### 4.1. Cell Culture and PAA Hydrogel-Coated Dishes 

Human gastric cancer MKN28 cells (a gift from Professor Yoshiaki Ito, Cancer Science Institue, Singapore) were used for the generation of CRISPR/Cas9 knock-out cells (YAP KO and TAZ KO) and control WT cells. MKN28 cells were cultured in RPMI-1640 medium (Nacalai Tesque, Kyoto, Japan), supplemented with 10% (*v*/*v*) heat-inactivated fetal bovine serum (FBS, Gibco, Thermo Fisher Scientific, Waltham, MA, USA) and 1% (*v*/*v*) penicillin-streptomycin (Pen-Strep, Gibco) in the incubator at 37 °C, 5% CO_2_. Mouse mammary gland epithelial EpH4-Ev cells were purchased from ATCC (ATCC CRL-3063) and YAP-null EpH4 cells were generated in our previous work [21]. EpH4 cells were cultured in DMEM medium (Gibco) supplemented with 10% (*v*/*v*) heat-inactivated FBS (Gibco, UA), 1% (*v*/*v*) Pen-Strep (Gibco), and 1.2 mcg/mL Puromycin (Gibco) in the incubator at 37 °C, 5% CO_2_.

PAA hydrogels with varying rigidity (0.5 kPa, 2 kPa, 4 kPa, 50 kPa, and 100 kPa) bound to 35 mm polystyrene dishes were purchased from Matrigen (Matrigen, Irvine, CA, USA). Some dishes were pre-coated with Collagen and the others were not coated with extracellular matrix (ECM). The non-ECM-coated hydrogel dishes were coated with Fibronectin according to the manufacturer’s instructions. For the comparison of cell growth and shape on the hydrogel dishes with various rigidity, the same number of cells were seeded and maintained for 2–3 days on the respective rigidity. For the prolonged culture of the cells on the soft conditions, 0.5 kPa hydrogel-coated dishes were used and the cells were maintained for 10–13 days. Subsequently, cell clusters were collected gently with a 5 mL serological pipette and transferred into multiple plastic dishes or fibronectin-coated glass-bottom dishes by diluting with complete culture medium. Cell attachment and spreading were monitored by counting the number of clusters in suspension and the number of clusters spreading on the substrate for the following 3–5 days.

### 4.2. Generation of CRISPR/Cas9 YAP and TAZ Knock-Outs and Overexpression of YAP or TAZ

Guide RNA (gRNA) sequences against human *YAP* (*hYAP* gRNA1: 5′-CATCAGATCGTGCACGTCCG, *hYAP* gRNA2: 5′-ATCAGATCGTGCACGTCCGC) and human *TAZ* (*hTAZ* gRNA1: 5′-CGGGTGGCCGCCCGACGAGT or *hTAZ* gRNA2: 5′-CAGCTCGTCGGTCACGTCGT) were designed using the online CRISPR design tool (http://crispr.mit.edu) and subsequently cloned into the GeneArt CRISPR Nuclease vector with orange fluorescent protein (OFP) reporter (Thermo Fisher Scientific, Waltham, MA, USA). WT MKN28 cells were then transfected with the CRISPR vectors using Lipofectamine2000 (Thermo Fisher Scientific) according to the manufacturer’s instructions. Three days post-transfection single cells expressing OFP were sorted into 96-well plates, expanded, and individually screened by Western blot for loss of YAP or TAZ protein expression, respectively.

A single knock out clone generated from each guide RNA (four cell lines in total) was subsequently selected. Knock out cells were transduced with a retrovirus carrying pBABE puro-empty vector, human YAP1-2δ^S127A^ or mouse TAZ^S89A^ coding DNA to generate stably transduced “active” YAP/TAZ over-expressing cells, or empty vector control cells. Cells were selected using 1 µg/mL puromycin (Thermo Fisher Scientific) for two weeks before YAP or TAZ over-expression was verified by Western blot.

### 4.3. Protein Isolation and Western Blot

Cells were washed with phosphate-buffered saline and incubated for one hour on ice in RIPA buffer (50 mM Tris-HCl (pH 7.3), 150 mM NaCl, 0.75 mM EDTA, 0.5% Sodium deoxycholate, 1% Triton X-100 supplemented with 1:100 protease inhibitor mixture (Sigma-Aldrich, St. Louis, MO, USA), 1:100 phosphotase inhibitor mixture (Sigma-Aldrich), 0.5 U/mL Benzonase Nuclease, 1 mM MgCl_2_). Total cell lysates were clarified by centrifugation before being boiled in sample buffer (2% (*w*/*v*) SDS, 10% (*v*/*v*) glycerol, 62.5 mM Tris pH 6.8, 0.02% (*w*/*v*) bromophenol blue, with 1% (*v*/*v*) 2-mercaptoethanol). 

Protein lysates (20 µg of total protein) were separated by SDS-PAGE using linear Tris-glycine gels in running buffer (25 mM Tris pH 8.3, 192 mM glycine, 0.1% SDS (*w*/*v*)) using the Mini-PROTEAN Tetra Cell system (Bio-Rad, Hercules, CA, USA). The Bio-Rad Precision Plus Protein All Blue Pre-Stained Standards (Bio-Rad) was used as the molecular weight reference. Separated proteins were transferred to Immun-Blot PVDF membranes (Bio-Rad) using the Mini Trans-Blot Cell system (Bio-Rad) at 90 V for 90 min at 4 °C in transfer buffer (25 mM Tris-base, 192 mM glycine, 20% (*v*/*v*) methanol) before being washed with Tris-buffered saline containing Tween-20 (TBST; 25 mM Tris pH 7.5, 150 mM NaCl, 0.1% (*v*/*v*) Tween-20) and then blocked with 5% (*w*/*v*) skim milk powder in TBST for 30 min at room temperature. Primary antibodies (anti-YAP (Cell Signaling Technology, Danvers, MA, USA, Cat# 14074), anti-TAZ (Cell Signaling Technology, Cat# 4883), anti-GAPDH (Cell Signaling Technology, Cat# 5174), anti-β-actin (Cell Signaling Technology, Cat# 3700)) were diluted using 5% (*w*/*v*) skim milk in TBST and incubated with the PVDF membrane overnight at 4 °C. Membranes were washed four times for 5 min each with TBST and then incubated with secondary antibody for 1 h at room temperature, before washing again with TBST. Signals were detected using WesternBright Quantum Western Blotting HRP Substrate (Advansta, San Jose, CA, USA). Uncropped images are available in Figures S1 and S2.

### 4.4. Immunofluorescent (IF) Staining and Confocal Microscopy

Cells were fixed using 4% paraformaldehyde (PFA) in PBS for 15 min at room temperature (RT) and subsequently rinsed with PBS twice. Cells were permeabilized for 15 min with 0.3% Triton X−100 in PBS. Blocking was done with 5% BSA and 0.3% Triton X−100 in PBS for at least 2 h at RT. Cells were incubated with required primary antibodies: anti-YAP (made in house, and Santa Cruz Biotechnology, Dallas, TX, USA, Cat# sc101199), anti-TAZ (BD Biosciences, Franklin Lakes, NJ, USA, Cat# 560235), anti-phosphorylated Paxillin at Tyr118 (Invitrogen, Carlsbad, CA, USA, Cat # 44-722G), diluted in AB dilution buffer (2% BSA and 0.2% Triton X−100 in PBS) overnight at 4 °C. Next, cells were washed with three different IF washing buffers for 15 min each: (i) 0.2% Triton X−100 in PBS, (ii) 0.2% Tween 20 in PBS, (iii) PBS. Then, cells were incubated with Alexa-flour-conjugated secondary antibodies and Phalloidin diluted in the AB dilution buffer for 1 h at RT, followed by washing with the three IF washing buffers for 15 min each. The nuclei were stained with NucBlue Live Ready Probes (Molecular Probes, Eugene, OR, USA) in PBS for 10 min at RT before imaging. The stained samples were scanned using the Nikon A1Rsi confocal microscope (Nikon, Tokyo, Japan) with a 100× objective for Paxillin staining or a 40× objective for staining the other proteins. The microscopy images were analyzed using ImageJ and Imaris software (Oxford Instruments, Oxfordshire, UK). Collages of the IF images are available in Figures S3, S4 and S8.

### 4.5. RNA Sample Preparation and RNA-seq

The cells at ~50% confluent in a 100 mm dish were collected and RNA was prepared with miRNeasy Mini Kit (QIAGEN, Hilden, Germany) according to the manufacturer’s protocol with the use of QIAshredder spin columns (QIAGEN) to homogenize the cell lysates. Quality check on the RNA samples, RNA-seq library preparation, sequencing, pre-processing and quantification of sequencing reads were done by AITbiotech (AITbiotech, Singapore, Singapore). Briefly, ribosomal RNA was depleted using Epicentre Ribo-zero rRNA Removal Kit (Epicentre, Madison, WI, USA). Then, sequencing libraries were prepared using NEBNext Ultra Directional RNA Library Prep Kit for Illumina (NEB, Ipswich, MA, USA) and subsequently sequenced using the Illumina HiSeq 2500 System. Paired-end reads were mapped onto the reference genome (Human GRCh37, release82) using TopHat2 (v2.0.9) [38]. The transcriptome assembly, and subsequent quantification of individual transcripts into the normalized read counts (e.g., Fragments Per Kilobase Million (FPKM) was done using Cufflinks and Cuffdiff (v2.1.1) [39]. The RNA-seq datasets from this study have been deposited in NCBI’s Gene Expression Omnibus (GEO) database under accession number GSE161631.

### 4.6. RNA-seq Data Analysis

The count data including normalized FPKM values of the 8 samples were imported into R [40]. A filter that at least two observations with absolute value ≥ 1 were applied to remove the transcripts with low expression, resulting in 31,649 transcripts from 83,942. FPKM + 1 values were used to calculate log_2_ fold change in expression between treatment (overexpression, OE) and control (endogenous expression, N) groups. If two biological replicates (KO clone 1 and 2, generated using different gRNA sequences) consistently showed a greater than the 2-fold difference (|log2$\frac{OE\left(KOclone1\right)}{N\left(KOclone1\right)}$| > 1 and |log2$\frac{OE\left(KOclone2\right)}{N\left(KOclone2\right)}$| > 1), the transcripts were defined as DETs. k-means clustering analysis was performed using Cluster3.0 [16] and heat map visualization was conducted using Java TreeView [41]. GO analysis and visualization via the GO enrichment map were implemented using clusterProfiler [19], a Bioconductor/R package.

### 4.7. RNA Isolation and Quantitative Reverse Transcription PCR (RT-qPCR) Assay 

The cells in the culture conditions of our interest were lysed by adding RLT+ buffer (QIAGEN) directly to the cells after aspirating the culture medium. Next, RNA was isolated using the RNeasy Plus Mini Kit (QIAGEN), and subsequently, cDNA was synthesized using SuperScript IV reverse transcriptase (Invitrogen, USA) according to the manufacturer’s protocols. RT-qPCR was performed using SsoAdvance Universal SYBR Green Supermix (Bio-Rad) and Bio-Rad CFX 96 Real-Time PCR Detection System according to the manufacturer’s protocols. The relative expression of a gene was calculated using the delta-delta Ct (ddCt) method, normalized with a housekeeping control gene, *HPRT1*, and the expression level in WT. The relative expression was 2^(−ddCt)^, formatted as a percentage. The qPCR primers were designed using NCBI Primer-BLAST and the primer sequences are listed in Table 1

## 5. Conclusions 

We conducted RNA-seq analysis of overexpressed YAP and TAZ, in the absence of its paralogous counterpart, to identify unique and common YAP- and TAZ-transcriptional targets in the gastric cancer cell line, MKN28. We identified the presence of common YAP/TAZ-driven transcriptional signatures in gastric cancer cells: activation of platelet degranulation and lipoprotein particles, which are closely associated with cancer cell growth, survival and metastasis. Moreover, we found that the cell-substrate junction GO term was specifically enriched for YAP transcriptional targets. We validated YAP’s specific gene signature in the regulation of cell-substrate junction by interrogating cell attachment and spreading of YAP KO, TAZ KO, and WT cells on hydrogel dishes with varying rigidity, and upon switching from very soft to stiff conditions. Our study identifies functional targets regulated solely by YAP or TAZ, and in common by both YAP and TAZ as potent transcriptional co-activators in gastric cancer cells. We also uncovered an essential function of YAP in the spreading of cancer cells onto the new rigid environments, which has important ramifications for understanding metastasis.

## Figures and Tables

**Table 1:** The qPCR primers were designed using NCBI Primer-BLAST and the primer sequences.

Gene	Forward	Reverse
*PARVA*	AGAAAGCCAAGGAGGTGTCCG	TCCAGCTCAAAGGGAATTGGG
*FHL2*	GCATTTTGACTTTGGGGTTGCT	AGATTCGTTGCAATGGTGGC
*AJUBA*	AGGTTCCAGCCCCTTCTCTAT	CTCCTGAAACCCTGAAAACAGAT
*BCAR1*	ATCCCTGCCTCAGAAACGTG	CACCGTCATGATGTCACCCT
*LIMS2*	CACCATGACGGGAAGCAATATGT	GCTGTTGACAATGCGCTCG
*DPP4*	GCCGACGATGAAGACACCG	TTTGTTCAGCAGAACCACGGG
*CYR61*	TCCCTGTTTTTGGAATGGAG	GAGCACTGGGACCATGAAGT
*CTGF*	TGCATCCGTACTCCCAAAAT	ATGTCTTCATGCTGGTGCAG
*HPRT1*	ATGGACAGGACTGAACGTCTT	TCCAGCAGGTCAGCAAAGAA

## Supplementary Materials

The following are available online at https://www.mdpi.com/2072-6694/12/12/3667/s1, Figure S1: Uncropped images of western blot assays (related to Figure 1A). Western blot analysis of YAP KO and TAZ KO clones generated via the CRISPR/Cas9 system using two independent guide RNA sequences (labelled as 1 and 2) against human YAP and human TAZ. The WT sample is shown in the first and last lane (Top). β-actin is used as a loading control (Bottom). The protein molecular weight size marker (unit: kDa) is depicted along the left of the Western blot images. The dotted red rectangle indicates the cropped portion of the image; Figure S2: Uncropped images of western blot assays (related to Figure 1C,D). Western blot analysis of YAP KO cells transduced with empty vector (EV, control) or TAZ^S89A^ (overexpression, OE) (Left top). GAPDH is used as a loading control (Left bottom). Western blot analysis of TAZ KO cells transduced with empty vector (EV) or YAP^S127A^ (OE), and β-actin is used as a loading control (Right). The dotted red rectangle indicates the cropped portion of the image; Figure S3: Collage of YAP IF images for MKN28 WT, YAP KO clone 1 and 2, and TAZ KO clone 1 and 2 cells (related to Figure 1B). YAP is shown in the pseudo-colour red. A scale bar = 30 µm; Figure S4: Collage of TAZ IF images for MKN28 WT, YAP KO clone 1 and 2, and TAZ KO clone 1 and 2 cells (related to Figure 1B). TAZ is shown in pseudo-colour green. A scale bar = 30 µm; Figure S5: RT-qPCR validation of the cell-substrate adhesion genes in YAP KO cells in comparison to WT cells. Error bars indicate standard error of the mean, calculated from four replicates. *CYR61* and *CTGF* are known as transcriptional targets of YAP and TAZ; Figure S6: (A) Representative microscopy images of Paxillin phosphorylated at Tyr118 (p-Paxillin in red) and DAPI (nucleus in turquoise) in WT and YAP KO MKN28 cells. Scale bar = 5 µm (B) Mean single FA volume, measured by p-Paxillin staining, in WT and YAP KO MKN28 cells. The red dot and black bracket indicate a mean and 95% confidence interval of the mean; Figure S7: (A) Representative images of WT and YAP KO EpH4 cells cultured in the typical tissue culture dish (Polystyrene), and polyacrylamide (PAA) hydrogel dishes with different rigidity (50 kPa and 4 kPa). (B and C) Western blot analysis of WT and YAP KO EpH4 cells cultured in different rigidity. β-actin is used as a loading control. The protein molecular weight size marker (unit: kDa) is depicted along the right of the Western blot images; Figure S8: Collage of phospho-Paxillin (Tyr118) images for MKN28 WT, YAP KO clone 1, and TAZ KO clone1 cells. Phospho-Paxillin (Tyr118) is shown in the pseudo-colour red along with DAPI (blue) and Phalloidin (green). A scale bar = 7 µm; Table S1: Top up-regulated and down-regulated transcriptional targets by YAP/TAZ in MKN28 cells; Video S1: (A) Phase-contrast live-cell images of spreading of MKN28 WT (Top) and YAP KO clone 1 (Bottom) cells, and (B) WT (Top) and TAZ KO clone 1 (Bottom) cells. Time-lapse images were taken every 15 min for 18 h. The actual timestamp is shown at the top left corner of the video. A scale bar = 20 µm.
