# Supplementary material for: Common and Unique Transcription Signatures of YAP and TAZ in Gastric Cancer Cells
Source: Cancers (Basel). 2020 Dec 7;12(12):3667. doi: 10.3390/cancers12123667 (PMC7762230; doi:10.3390/cancers12123667)

Supplementary Figure S1

Related to Figure 1A

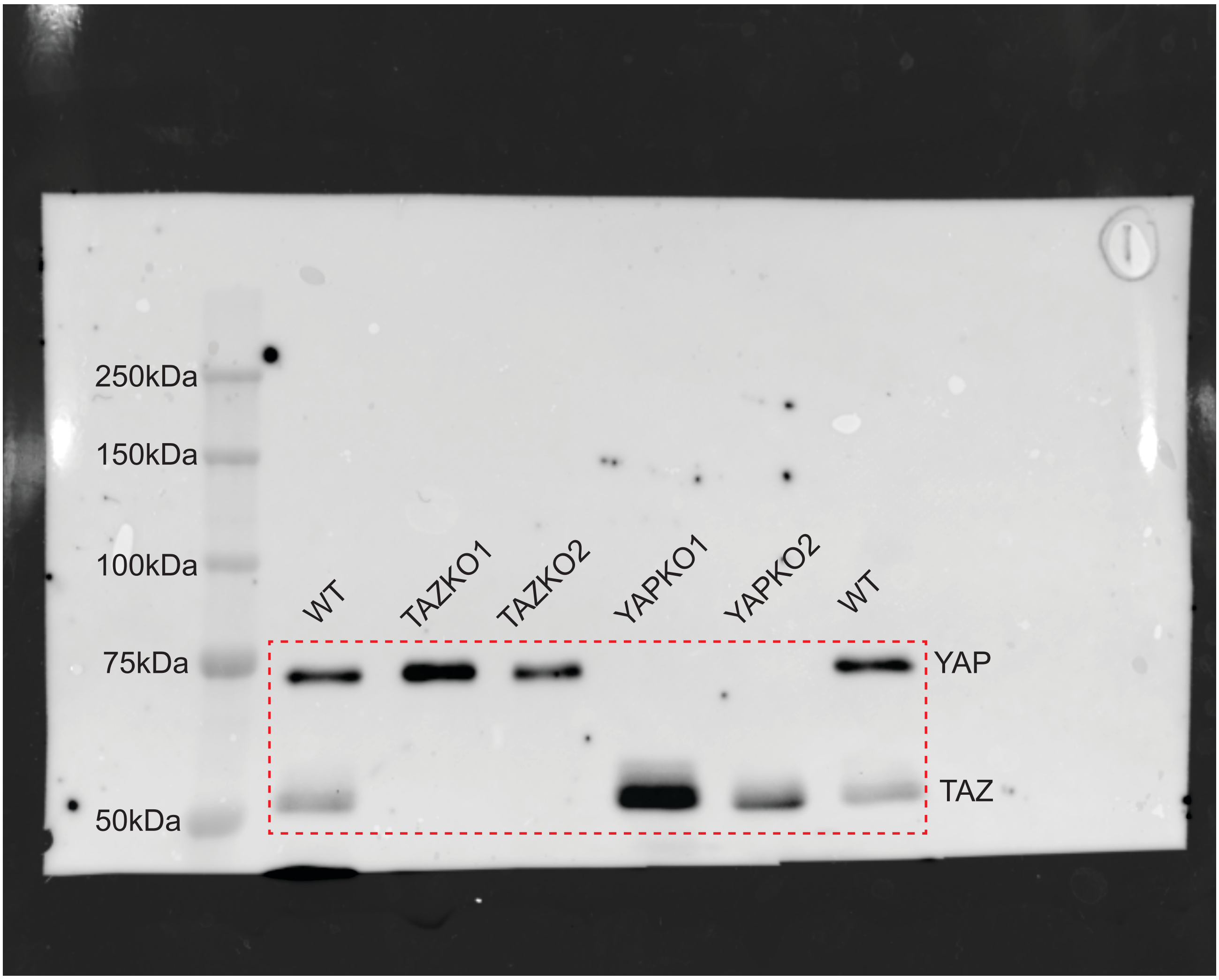

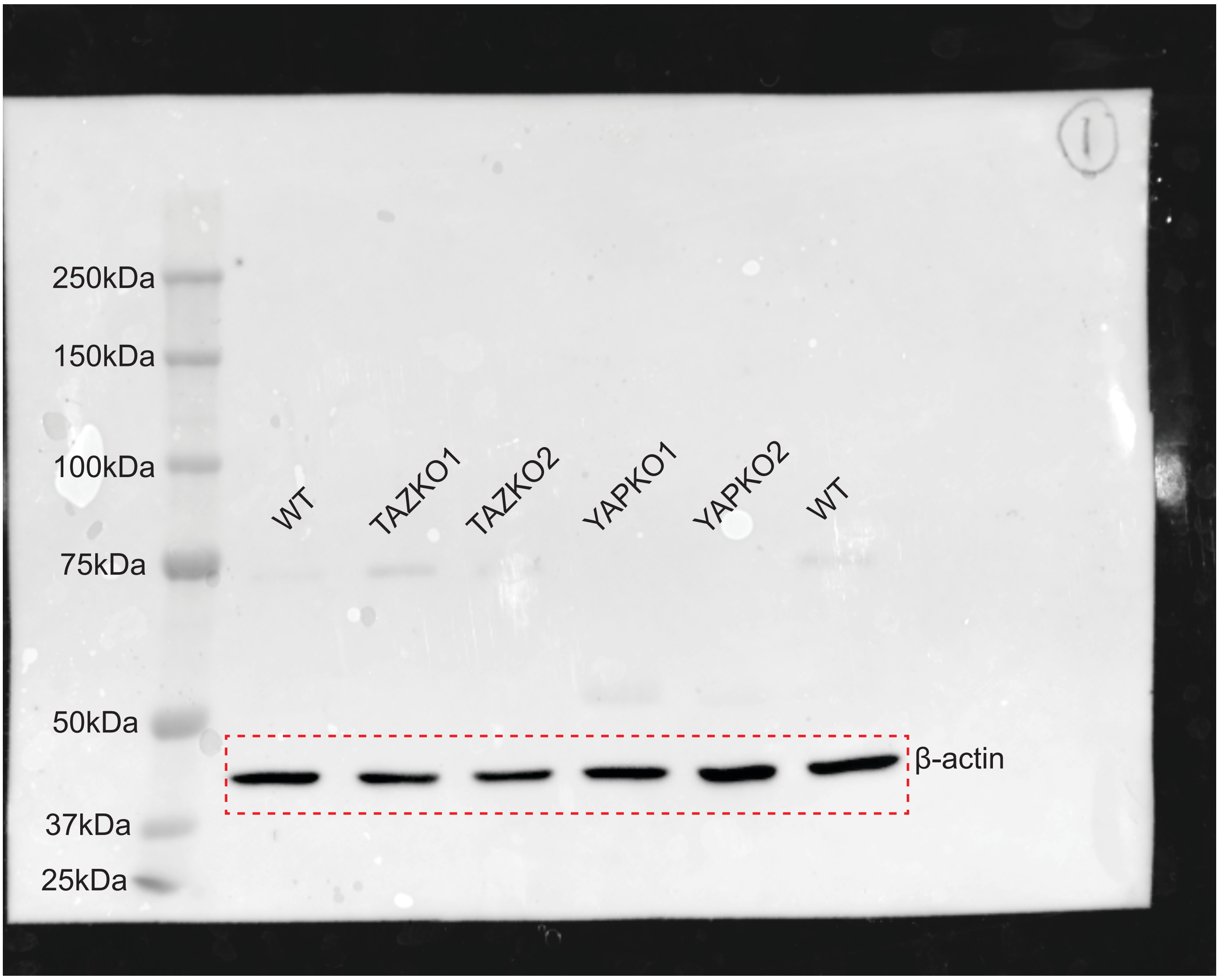

Supplementary Figure S2

Related to Figure 1C

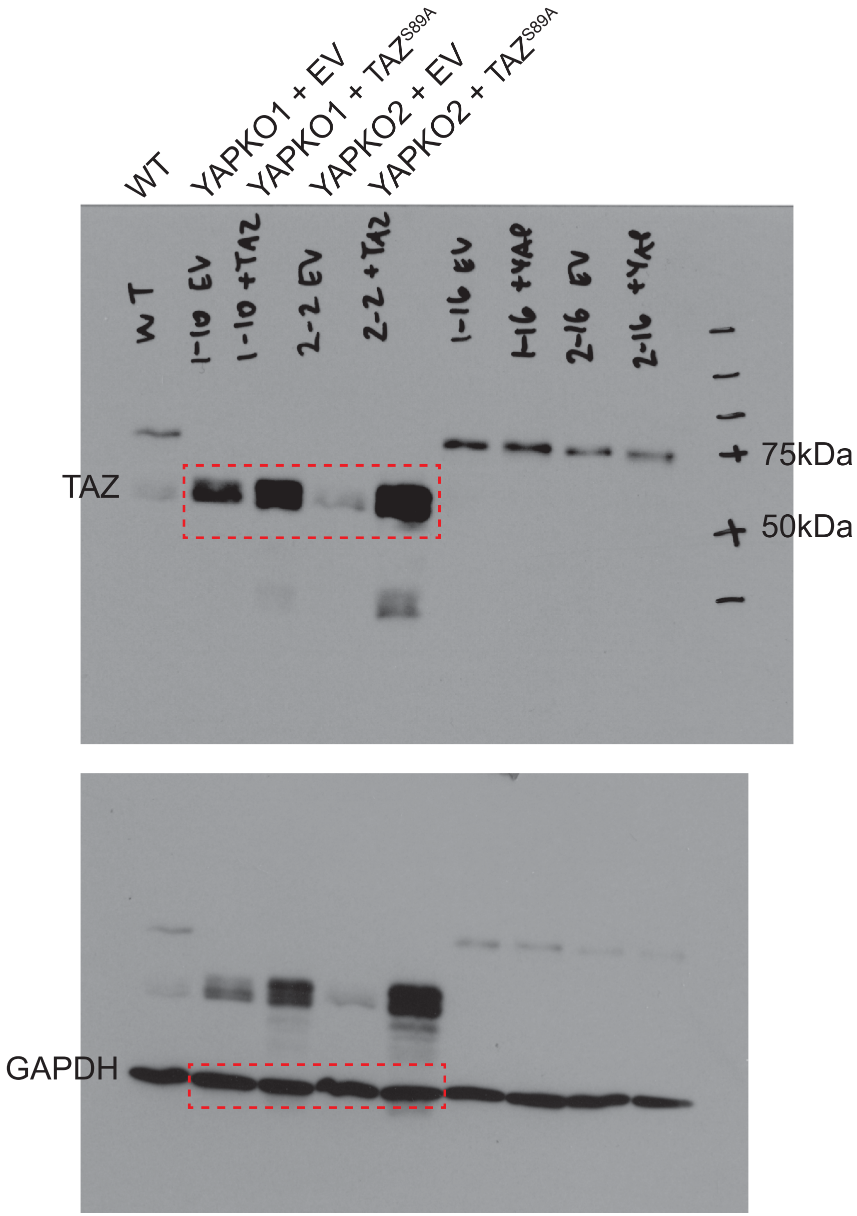

Related to Figure 1D

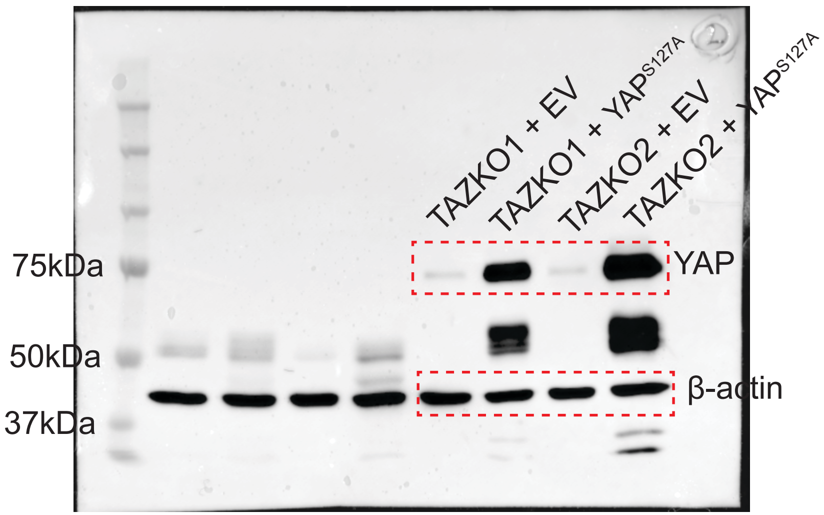

Supplementary Figure S3

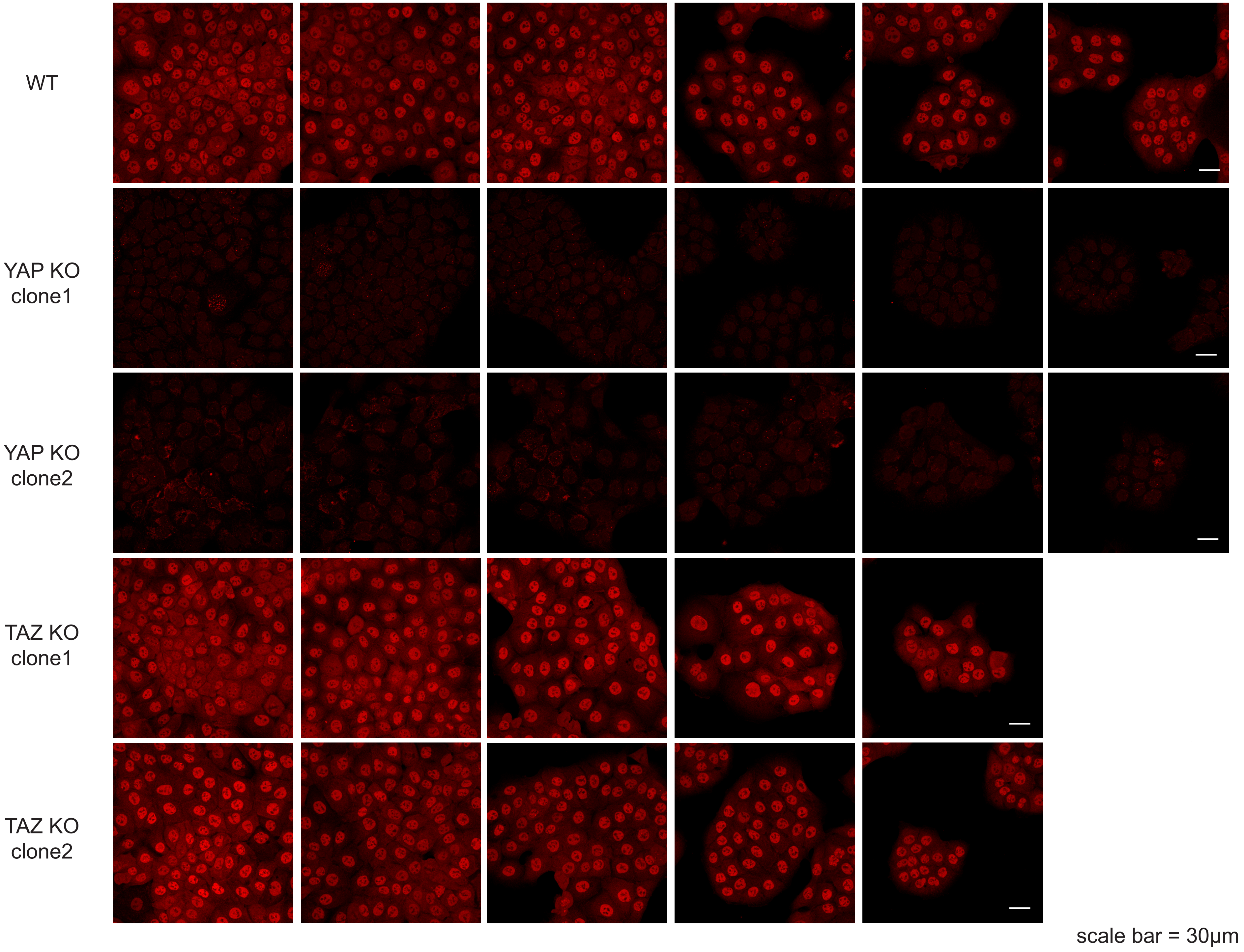

Supplementary Figure S4

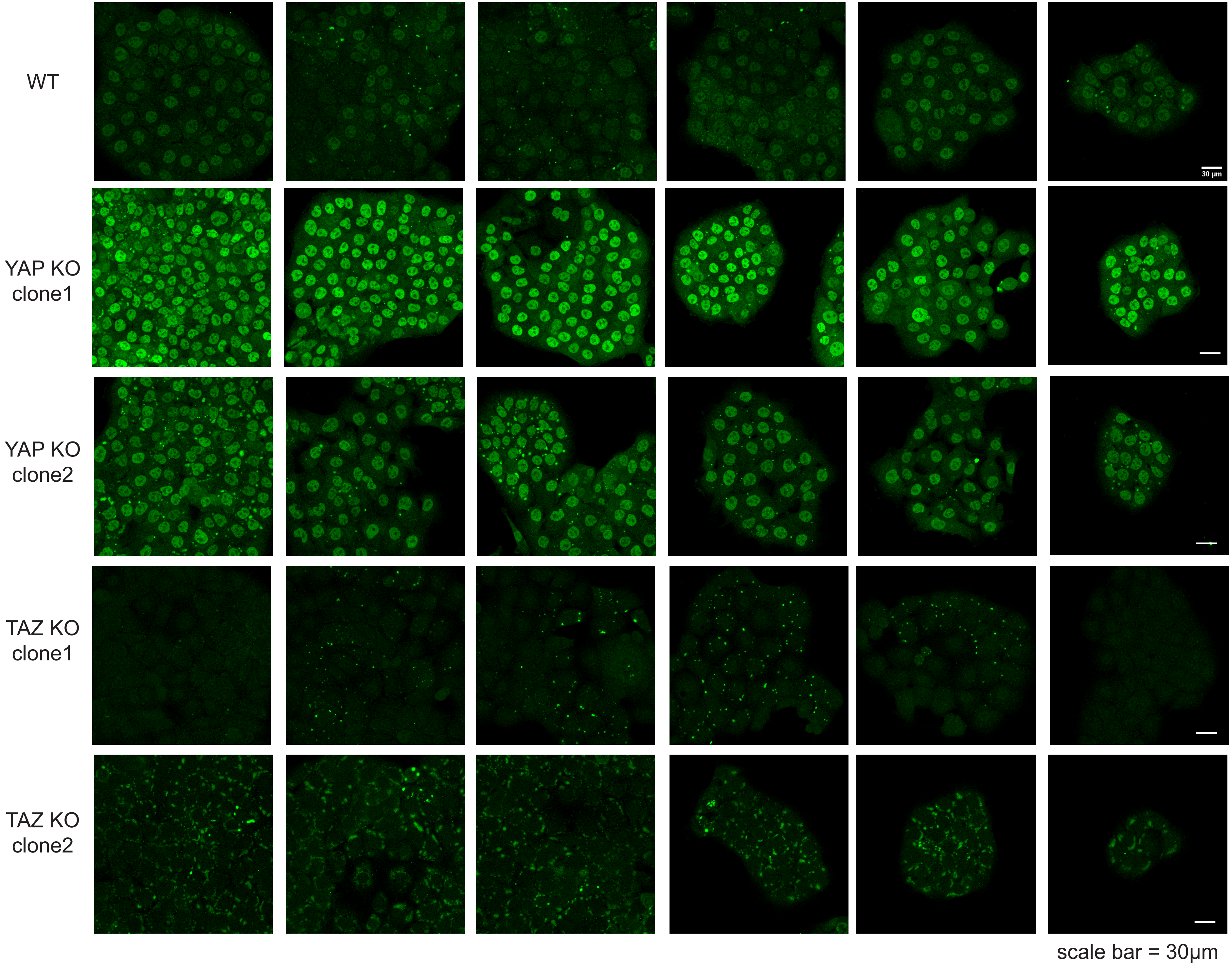

Supplementary Figure S5

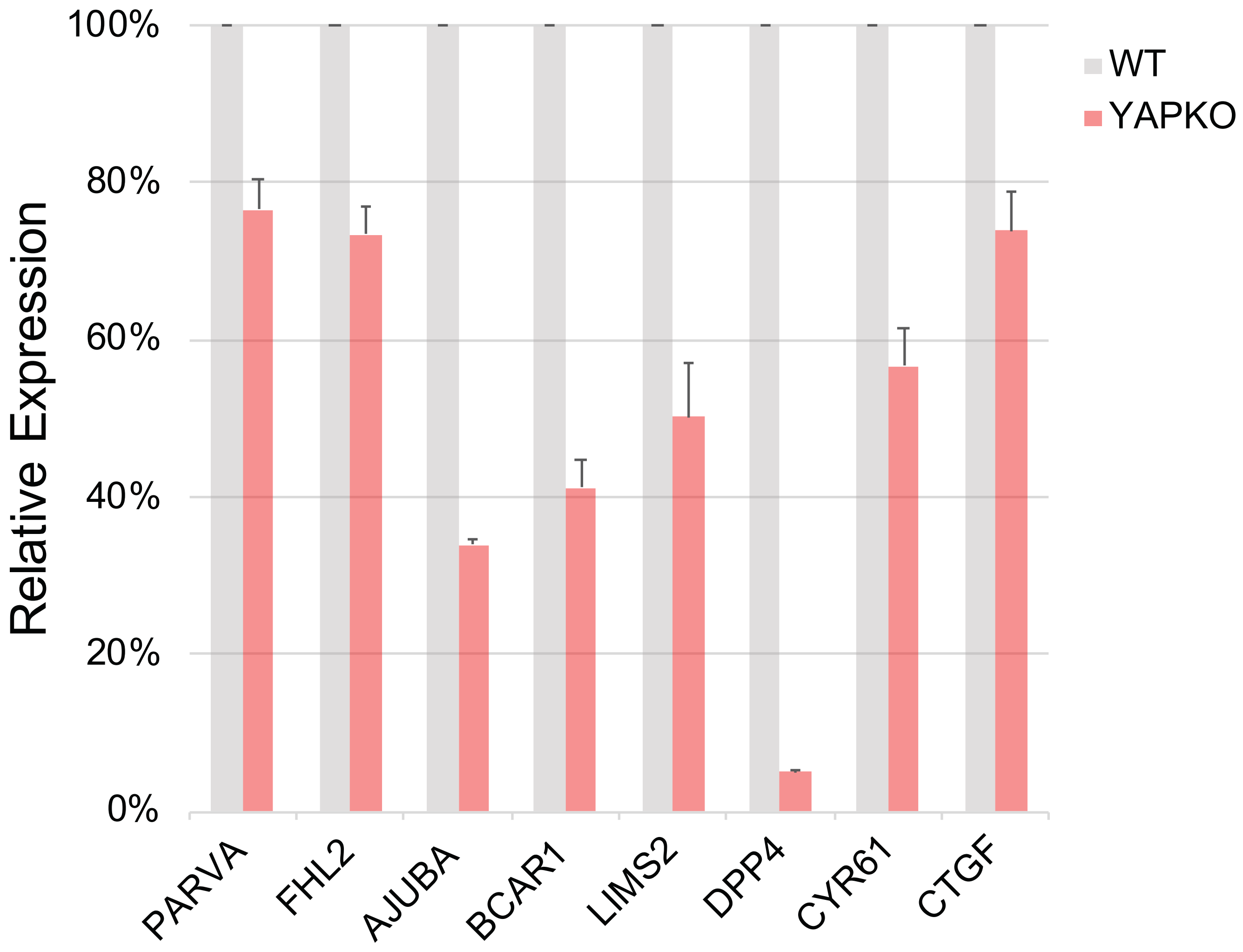

Supplementary Figure S6

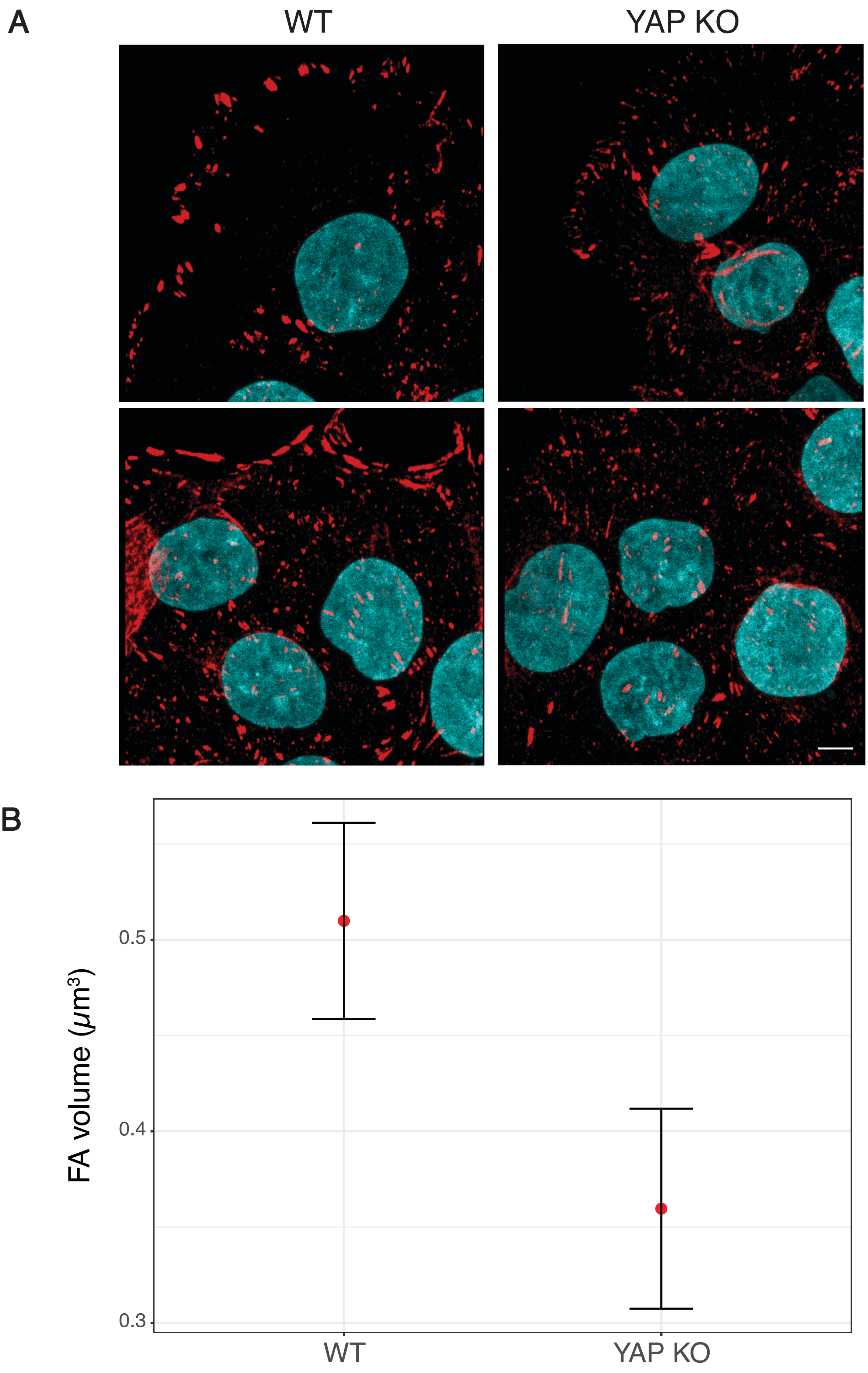

Supplementary Figure S7

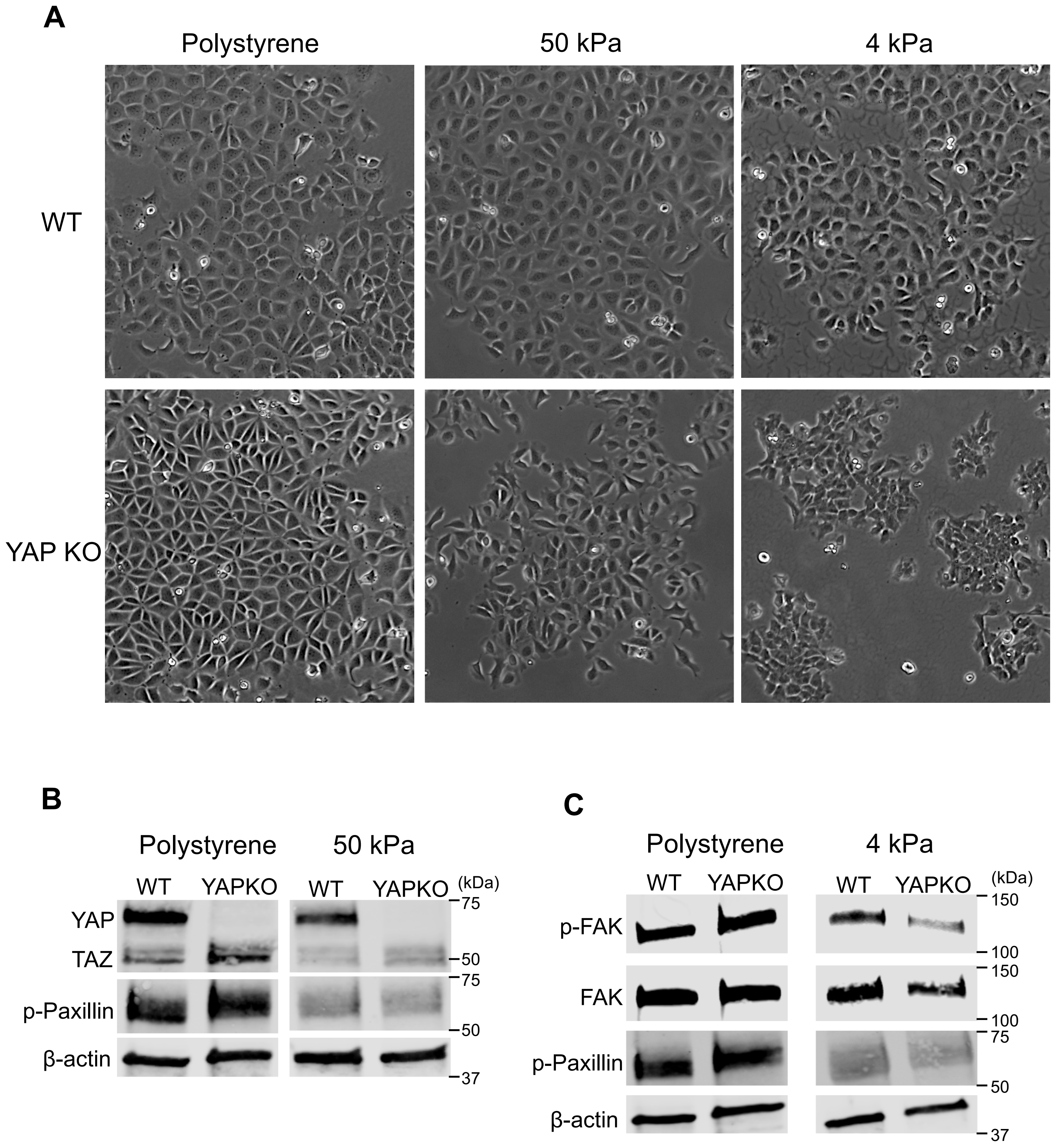

Supplementary Figure S8

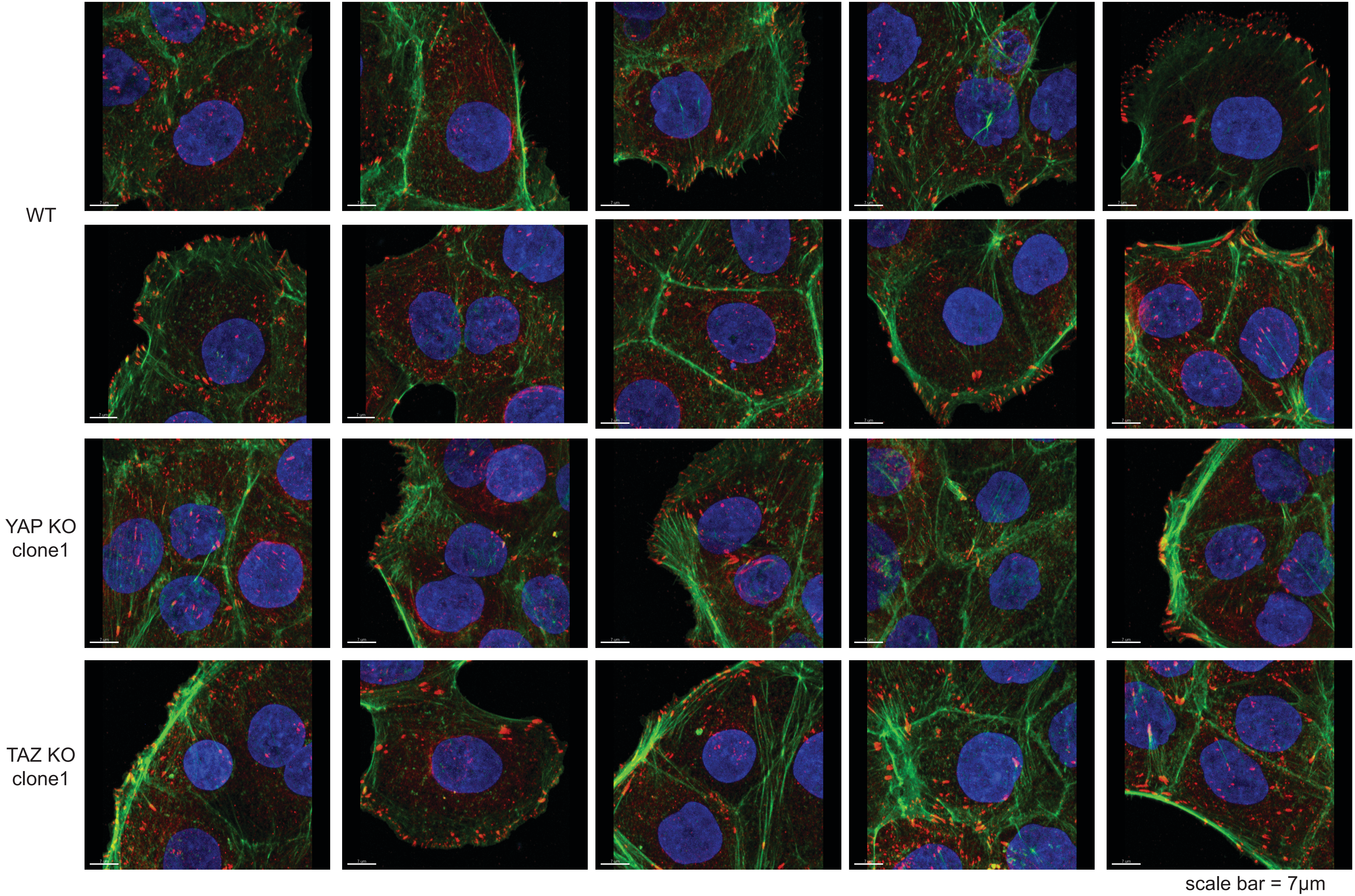

Supplement: Supplementary file 1 [file cancers-12-03667-s001.zip › final supp/cancers-982574 supp figures.pdf]
